# (Dis)agreement and concordance of metabolic indices from the oral glucose tolerance test and mixed‐meal tolerance test: Implications for application

**DOI:** 10.1113/EP093596

**Published:** 2026-06-17

**Authors:** Nina Sloth Nielsen, Nikolaj Agerlin, Nikolas Antoniou, Helga Ellingsgaard, Kristian Karstoft, Louise Lang Lehrskov, Grit Elster Legård, Mark Preben Printz Lyngbæk, Beckey Trinh, Thomas P. J. Solomon, Marc Y. Donath, Mathias Ried‐Larsen, Cody Garett Durrer

**Affiliations:** ^1^ Centre for Physical Activity Research Rigshospitalet Copenhagen Denmark; ^2^ Department of Clinical Pharmacology Bispebjerg and Frederiksberg Hospital University of Copenhagen Copenhagen Denmark; ^3^ Department of Clinical Medicine University of Copenhagen Copenhagen Denmark; ^4^ Department of Oncology Copenhagen University Hospital – Herlev and Gentofte Herlev Denmark; ^5^ Department of Rheumatology Department of Internal Medicine 2 Holbæk Hospital Holbæk Denmark; ^6^ Clinic of Endocrinology, Diabetes and Metabolism University Hospital Basel Basel Switzerland; ^7^ Blazon Scientific Brighton East Sussex UK; ^8^ Division of Endocrinology Department of Medicine Centre Hospitalier de l'Université de Montréal, University of Montréal Hospital Research Center Montreal Quebec Canada

**Keywords:** insulin resistance, insulin secretion, mixed‐meal tolerance test, oral glucose tolerance test, type 2 diabetes

## Abstract

Metabolic indices derived from the oral glucose tolerance test (OGTT) and mixed‐meal tolerance test (MMTT) are often interpreted interchangeably; however, these tests represent distinct physiological stimuli. In order to examine potential differential metabolic responses to these tests, we characterized the (dis)agreement between OGTT‐ and MMTT‐derived indices of insulin sensitivity, insulin secretion and β‐cell function. We also described their convergent validity in individuals with normal glucose tolerance (NGT) or type 2 diabetes (T2D). We conducted a *post hoc* analysis of eight trials with participants who underwent either OGTT or MMTT, or both. Insulin sensitivity, insulin secretion and β‐cell function were derived. Agreement was assessed using Bland–Altman regression approaches to estimate systematic and proportional bias and 95% limits of agreement. Linear mixed‐effects models were used to estimate means by glycaemic status (NGT vs. T2D), and area under the receiver operating characteristic (AUROC) curve analyses were used to evaluate discriminative ability between glycaemic status for the respective test indices. No systematic bias was observed. Proportional bias was present for insulin secretion and β‐cell function, with increasingly negative differences between methods (OGTT–MMTT) as outcome values increased. All indices exhibited relatively wide 95% limits of agreement, which widened further at higher magnitudes. The OGTT‐ and MMTT‐derived means were significantly different between NGT and T2D. We did not observe differences in AUROC between tolerance tests. Agreement between OGTT‐ and MMTT‐derived indices worsened as the respective value increased, suggesting differential stimulus–response characteristics in insulin secretion between tolerance tests. Despite this, descriptive analyses suggest that the test‐specific indices demonstrate convergent validity.

## Abstract (highlights)

- **What is the central question of this study?** How do metabolic indices derived from oral glucose and mixed‐meal tolerance tests compare, and what are the implications for test selection?
- **What is the main finding and its importance?**
  Observed methodological agreement between oral glucose tolerance test‐ and mixed‐meal tolerance test‐derived indices of insulin sensitivity, secretion and β‐cell function was poor, and proportional bias increased with outcome magnitude for insulin secretion and β‐cell function. Both tolerance tests display convergent validity in assessment of these physiological constructs (i.e., they preserve directional discrimination between normal glucose tolerance and type 2 diabetes). The distinct physiological response to each tolerance test emphasises the importance of choosing the right test for the specific research/clinical question.

## 1 INTRODUCTION

Insulin sensitivity has long been recognized as a key determinant of metabolic function (Himsworth, 1936). Various methods have been developed to assess insulin sensitivity, with the hyperinsulinaemic–euglycaemic clamp established as the gold standard (Defronzo et al., 1979). Although the hyperinsulinaemic–euglycaemic clamp remains the most direct method of assessment, it is invasive, labour intensive and costly, all of which limit its use in large‐scale or routine settings. It also relies on supraphysiological insulin levels and glucose flux, which do not reflect physiological regulation during daily living. Moreover, clamps and other intravenous tests bypass the gastrointestinal tract and therefore do not capture effects such as incretin stimulation (Holst, 2019). These limitations have led to widespread use of simpler indirect indices of insulin sensitivity, insulin secretion and β‐cell function.

Among these, the oral glucose tolerance test (OGTT) is established in both clinical practice and research. It is used for diagnosis of type 2 diabetes (T2D), investigating related conditions, such as metabolic syndrome, and to derive indirect indices of insulin sensitivity, secretion and β‐cell function. The Matsuda insulin sensitivity index (Matsuda ISI) is one such example of a widely applied and accessible measure of insulin sensitivity that can be derived from a standard 2‐hour OGTT (Matsuda & DeFronzo, 1999). Despite these benefits, as a glucose‐only challenge the OGTT does not capture the complexity of a typical meal. In contrast, a mixed‐meal tolerance test (MMTT), which contains carbohydrate, fat and protein, more closely mimics everyday food intake. As such, it elicits an arguably more ecologically relevant physiological response.

Despite these theoretical advantages, the MMTT remains comparatively underused in research and clinical settings. One barrier is the lack of standardization in meal composition, which varies widely across studies (Lages et al., 2022) and complicates cross‐comparison with OGTT‐derived outcomes. As a result, the expected response to a given MMTT protocol is often not well documented. Previous studies have compared glycaemic responses between OGTTs and MMTTs, but most have focused narrowly on diagnostic agreement using 2‐hour glucose concentrations (Chanprasertpinyo et al., 2017; Forbes et al., 2018; Marena et al., 1992; Meier et al., 2009; Newman et al., 2022; Selimoglu et al., 2009; Wolever et al., 1998; Wopereis et al., 2017). Despite their potential to provide deeper physiological insights, these investigations fall short of a comprehensive comparison. The few studies that compare OGTT‐ and MMTT‐derived indices (Rijkelijkhuizen et al., 2009; Selimoglu et al., 2009; Wopereis et al., 2017) report only simple correlations; these describe association within samples but do not directly assess methodological agreement (Carstensen, 2010). Established approaches, such as Bland–Altman analyses, can quantify the magnitude of bias and error to describe how the methods differ over the range of the outcome values. These quantities can provide insights into the underlying physiological responses and inform on the appropriateness of the respective tolerance test for a given application.

In addition, most prior work emphasised similarity between tests rather than disagreement. Yet in this context, the degree and nature of methodological disagreement is arguably more important. The OGTT and MMTT are not expected to be interchangeable, otherwise the rationale for the MMTT as a more physiologically valid challenge is invalid. Specifically, the pattern of methodological disagreement can provide insight into the different physiological responses to each respective tolerance test. Beyond agreement, it is also necessary to assess whether indices from each test are credible in their intended contexts of assessing metabolic function (i.e., insulin sensitivity, insulin secretion and β‐cell function). In this sense, both OGTT‐ and MMTT‐derived indices should distinguish between normal glucose tolerance (NGT) and T2D. Furthermore, they should produce the expected physiological patterns, such as the inverse relationship between insulin sensitivity and secretion. In other words, they should display convergent validity.

As such, the primary aim of this investigation was to investigate potential differential metabolic responses to the OGTT and MMTT by describing the (dis)agreement between OGTT‐ and MMTT‐derived indices of insulin sensitivity, insulin secretion and β‐cell function. In addition, we sought to evaluate the convergent validity (i.e., whether the tolerance tests provide consistent conclusions about the underlying index, regardless of different numerical values) of these indices in their respective contexts by: (1) comparing test‐specific estimated means between individuals with NGT and T2D; (2) determining the ability of the tolerance tests to discriminate between glycaemic states; and (3) confirming the expected inverse relationship between insulin sensitivity and secretion in the MMTT. This thorough exploratory investigation into the similarities and differences between the two tolerance tests will help to guide future research regarding proper test choice, given the specific aims and context of the investigation.

## 2 MATERIALS AND METHODS

### 2.1 Ethical approval

All studies were approved by the Scientific Ethics Committee of the Capital Region of Denmark (H‐16018062, H‐4‐2013‐098, H‐3‐2012‐141, H‐1‐2014‐060, H‐15008542, H‐1‐2010‐027 and H‐1‐2013‐059) and conducted in accordance with the principles of the *Declaration of Helsinki*. Written informed consent was obtained from all participants.

### 2.2 Study design

This was a descriptive *post hoc* analysis using baseline data from eight independent clinical trials conducted at the Center for Physical Activity Research (Table S1; Ellingsgaard et al., 2020; Lehrskov et al., 2018; Lehrskov et al., 2019; Karstoft et al., 2014a, 2014b; Müller et al., 2017; Jakobsen et al., 2016). As such, no prespecification of the statistical analysis or outcome assessments was performed. These trials assessed glycaemic control using either the MMTT alone (two of the eight studies), OGTT alone (one of eight), or both the MMTT and OGTT in the same participants (five of eight). The paired dataset is used to address the primary and secondary aims of this study, whereas the unpaired dataset is included to estimate representative glucose and insulin curves for the different tests. A summary of included studies, detailing participant characteristics, test procedures and references to original publications, is provided in Table S1. Protocols for conducting the tolerance tests were standardized within the research centre (i.e., across studies); however, the composition of the MMTTs was not standardized fully across studies (Table S2). Importantly, the total calories and fat/protein content differed between NGT studies and T2D studies. Furthermore, owing to differences in the timing of blood collection across studies and the lack of paired OGTT and MMTT in some studies, subsets of the combined data were used for each objective (detailed below).

### 2.3 Study population

Participants were identified as either NGT (glycosylated haemoglobin <39 mmol/mol; American Diabetes Association Professional Practice Committee 2025) or T2D (previously diagnosed by a physician). Participant characteristics for each group are shown in Table 1. All participants with T2D who were included in the agreement analysis (studies 5–8) discontinued glucose‐lowering medication usage 48 hours prior to the tests. Participants were also instructed to avoid vigorous exercise within 48 hours prior to the tolerance tests. The use of glucose‐lowering medication for each study is reported in Table S1.

**TABLE 1:** Subject characteristics by glucose tolerance classification.

Characteristic	NGT (*n* = 72)	T2D (*n* = 30)
Sex, female/male (%/%)	23/49 (32%/68%)	19/11 (63%/37%)
Age, years	40 [29 to 51]	65 [56 to 68]
HbA1c, mmol/mol	34.9 ± 3.4	46.9 ± 6.4
Body mass, kg	93.8 [80.1 to 103.6]	91.2 [82.6 to 102.6]
Body mass index, kg/m^2^	31.8 ± 5.6	30.3 ± 4.5
T2D duration, years	–	6.5 [4.0 to 9.0]
Fasting glucose, mmol/L	4.9 [4.6 to 5.2]	7.5 [6.7 to 8.7]
Fasting insulin, pmol/L	78.2 [52.0 to 106.0]	98.6 [62.7 to 147.9]

*Note*: Data are summarized for the pooled datasets used in the agreement and convergent validity analyses. Counts are presented as *n* (%) and continuous outcomes as the mean ± SD or median [25^th^–75^th^ percentile], depending on the empirical data distribution.

Abbreviations: HbA1c, glycosylated haemoglobin; NGT, normal glucose tolerance; T2D, type 2 diabetes mellitus.

### 2.4 Experimental procedure

In the morning after an overnight fast of ≥8 hour, participants underwent either an MMTT or an OGTT. For both NGT and T2D participants, the MMTT contained ∼62 g of carbohydrate, although the amount of protein, fat and total caloric intake were different between protocols. Details of the mixed‐meal caloric and macronutrient composition are reported in Table S2. For the OGTT, participants consumed 300 mL of a glucose solution containing 83 g dextrose monohydrate (equivalent to 75 g anhydrous glucose; 300 kcal). Both the MMTT and OGTT drinks were served chilled. In both tests, venous blood samples were collected over the subsequent 2 hours (0, 15, 30, 60, 90 and 120 minutes) for determination of glucose and insulin. Glucose and insulin were analysed at the Department of Clinical Biochemistry, Rigshospitalet, using standard procedures.

### 2.5 Calculations for different surrogate indices for insulin sensitivity and secretion

Plasma glucose and insulin concentrations were used to calculate insulin sensitivity, insulin secretion and β‐cell function as follows.

Insulin sensitivity index (glucose was converted and expressed in milligrams per decilitre, with insulin in micro‐units per millilitre):

Matsuda ISI (Matsuda & DeFronzo, 1999) = $10000/(\sqrt{\mathrm{Insuli}n_{\mathrm{mean}}\;\times \;\mathrm{Glucos}e_{\mathrm{mean}}\;\times \;\mathrm{Insuli}n_{\mathrm{fasting}}\;\times \;\mathrm{Glucos}e_{\mathrm{fasting}}})$.

Insulin secretion index (glucose is converted and expressed in milligrams per decilitre, with insulin in micro‐units per millilitre):

AUC_ins/glu_ (Ahrén & Pacini, 2004) = ($\mathrm{Total}\;\mathrm{area}\;\mathrm{under}\;\mathrm{the}\;\mathrm{curv}e_{\mathrm{insulin},\;0-120\;\min}$)/($\mathrm{Total}\;\mathrm{area}\;\mathrm{under}\;\mathrm{the}\;\mathrm{curv}e_{\mathrm{glucose},\;0-120\;\min}$).

β‐Cell function, defined as the disposition index (DI):

DI (Bergman et al., 1981; Retnakaran et al., 2008) = $\mathrm{Matsuda}\;\mathrm{ISI}\times \mathrm{AU}C_{\mathrm{ins}/\mathrm{glu}}$.

### 2.6 Statistical analyses

The descriptive baseline characteristics of participants are presented as either the observed mean with SD (if normally distributed) or the median with 25th and 75th percentile (if non‐normally distributed) for each respective group. All statistical analyses were conducted in R (v.4.3.3; R Core Team, 2024). Statistical significance was defined as *P* < 0.05. No a priori power calculation was performed owing to the *post hoc* nature of this analysis.

#### 2.6.1 Agreement between assessment methodologies

**FIGURE 1:**
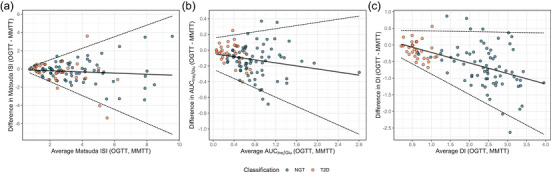
Agreement between OGTT and MMTT indices. Bland–Altman plots for Matsuda ISI (a), AUC_ins/glu_ (b) and DI (c), showing differences between OGTT and MMTT values against their average. Continuous lines indicate mean bias; dashed lines show 95% limits of agreement. NGT = blue and T2D = orange. Abbreviations: AUC_ins/glu_, insulin secretion index; DI, disposition index; Matsuda ISI, Matsuda insulin sensitivity index; MMTT, mixed‐meal tolerance test; NGT, normal glucose tolerance; OGTT, oral glucose tolerance test; T2D, type 2 diabetes mellitus.

Agreement was evaluated via methods described by Bland and Altman. Specifically, the difference between methods was regressed on their average (model 3.1; Bland & Altman, 1999), allowing for the quantification of both systematic and proportional bias (Figure 1). In this model, the intercept term (systematic bias) reflects a systematic difference between methods that is constant across the range of the observed data. The slope term (proportional bias) reflects the linear relationship between the difference in methods and the magnitude of the outcome measure (e.g., the difference might grow larger at higher values). In addition, the 95% limits of agreement (LoA) were calculated using model 3.3 (Bland & Altman, 1999), again allowing the LoA to change linearly with the magnitude of the outcome value. For a standardized metric of agreement, Lin's concordance correlation coefficient (CCC) was calculated, comparing OGTT versus MMTT for each index (DescTools v.0.99.60; Signorell, 2025). Indices from both OGTTs and MMTTs from studies 1, 3 and 5–7 were used (Table S1) to assess agreement.

#### 2.6.2 Estimated means for T2D and NGT using the MMTT versus OGTT

To assess the convergent validity of the MMTT and OGTT as tools for characterizing glycaemic control descriptively, we initially examined whether each test would provide statistically different mean estimates of the Matsuda ISI, AUC_ins/glu_ and DI for the different glycaemic classifications (NGT vs. T2D). A linear mixed‐effects model was used, with classification (NGT vs. T2D), test type (MMTT vs. OGTT) and their interaction (classification × test) as fixed effects and with participant identity as a random effect (lme4 v.1.1‐37; Bates et al., 2015, 2025). The Matsuda ISI was log‐transformed before analysis to meet model assumptions of normality and homoscedasticity. Estimated marginal means and mean differences were back‐transformed in this case (with mean differences presented as percentage differences). Pairwise contrasts estimating the mean differences between classification within each test type were performed to determine whether the estimated means for each index were different for NGT vs. T2D for each test type (emmeans v.1.11.1; Lenth, 2025). Indices from both OGTTs and MMTTs from studies 1, 3 and 5–7 were used (Table S1).

#### 2.6.3 Discriminative ability of OGTT versus MMTT for estimating T2D versus NGT

**FIGURE 2:**
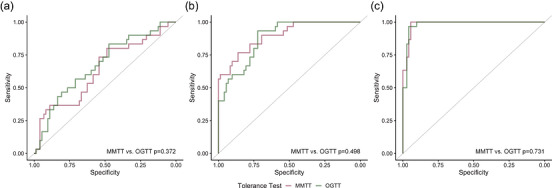
Receiver operating characteristic curves comparing OGTT and MMTT performance for classifying glycaemic status. Classification performance for Matsuda insulin sensitivity index (a), insulin secretion index (b) and disposition index (c). *P*‐values are shown for the difference between OGTT versus MMTT. Abbreviations: MMTT, mixed‐meal tolerance test; OGTT, oral glucose tolerance test.

For further evidence of convergent validity, we assessed the discriminative performance of OGTT‐ and MMTT‐derived indices using receiver operating characteristic (ROC) analyses (pROC v.1.18.5; Robin et al., 2011, 2023) to assess how well the Matsuda ISI, AUC_ins/glu_ and DI predicted glycaemic status (NGT vs. T2D) (Figure 2). For each test and index, we report area under the receiver operating characteristic (AUROC) curve and *P*‐values for the difference in AUROC between test types (calculated via DeLong's test). Indices from both OGTTs and MMTTs from studies 1, 3 and 5–7 were used (Table S1).

#### 2.6.4 Reciprocal relationship between insulin sensitivity and secretion

**FIGURE 3:**
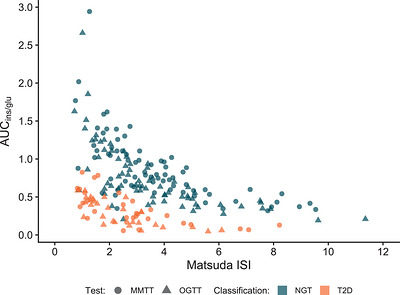
Rectangular hyperbolic relationship between insulin sensitivity and insulin secretion derived from OGTT (triangles) and MMTT (circles) in participants with NGT (blue) and T2D (orange). Abbreviations: AUC_ins/glu_, insulin secretion index; Matsuda ISI, Matsuda insulin sensitivity index; MMTT, mixed‐meal tolerance test; NGT, normal glucose tolerance; OGTT, oral glucose tolerance test; T2D, type 2 diabetes mellitus.

To explore whether the MMTT‐derived indices of insulin sensitivity (Matsuda ISI) and secretion (AUC_ins/glu_) display the expected proportionate and reciprocal relationship to each other (Retnakaran et al., 2008), a scatter plot of individual‐level data derived from both OGTT and MMTT is presented (Figure 3). Data were stratified by test type (OGTT vs. MMTT) and glycaemic status (NGT vs. T2D) to visualize potential group‐ and test‐specific patterns. Indices from both OGTTs and MMTTs from studies 1, 3 and 5–7 were used (Table S1).

#### 2.6.5 Glucose and insulin time–concentration curves

Representative time–concentration curves for glucose and insulin for NGT and T2D were generated using data from each test, where all six time points were available. These curves are presented descriptively to illustrate the temporal dynamics of glycaemic and insulinaemic responses across populations and test types. Additionally, the area under the curve (AUC) for glucose and insulin was calculated using the trapezoid method. These AUCs were compared using a mixed‐effects linear model, with classification (NGT vs. T2D), test type (MMTT vs. OGTT) and their interaction (classification × test) as fixed effects and with participant identity as a random effect (lme4 v.1.1‐37; Bates et al., 2015, 2025) to describe potential differences in the response to each test within each glycaemic classification. Data from OGTTs and MMTTs with six collection time points from studies 1–8 were used (Table S1).

## 3 RESULTS

### 3.1 Agreement between OGTT and MMTT across indices

Systematic bias (i.e., intercept ≠ 0) was not observed for Matsuda ISI (0.071 [95% confidence interval (CI): −0.597 to 0.455]), AUC_ins/glu_ (−0.045 [95% CI: −0.111 to 0.022]) or DI (0.089 [95% CI: −0.130 to 0.309]) (Figure 1). However, as indicated by the significant negative slope (−0.097 [95% CI: −0.180 to −0.014]), proportional bias was observed for AUC_ins/glu_ and was most pronounced for the DI (−0.317 [95% CI: −0.420 to −0.213]), whereas the association was negative but not statistically significant for Matsuda ISI (−0.063 [95% CI: −0.200 to 0.073]). In all cases, the difference between the two methods was larger as the magnitude of the outcomes increased, with the MMTT‐derived index being larger than the OGTT‐derived index. All indices exhibited wide LoA relative to the outcome values.

Absolute agreement assessed via CCC was 0.78 [95% CI: 0.69 to 0.84] for Matsuda ISI, 0.88 [95% CI: 0.84 to 0.92] for AUC_ins/glu_ and 0.74 [95% CI: 0.66 to 0.80] for DI.

### 3.2 Estimated glycaemic classification means

Estimated marginal means for Matsuda ISI, AUC_ins/glu_ and DI were consistently higher in NGT compared with T2D across all indices and both test types (Table 2). Mean contrasts confirmed significantly different index means by glycaemic classification (T2D vs. NGT; all *P* < 0.05), with the largest absolute difference observed for the DI. Comparisons between OGTT and MMTT revealed that the MMTT produced significantly larger group differences for AUC_ins/glu_ (0.09 [95% CI: 0.02 to 0.17], *P* = 0.018) and the DI (0.50 [95% CI: 0.25 to 0.74], *P* < 0.001], whereas ∆Matsuda ISI did not differ between tests (0.97 [95% CI: 0.84 to 1.12], *P* = 0.682).

**TABLE 2:** Estimated marginal means for OGTT and MMTT‐derived indices.

Estimated marginal means			
Variable	NGT (mean [95% CI])	T2D (mean [95% CI])	T2D–NGT (mean ∆ [95% CI]; *P*‐value)
**Matsuda ISI**			
MMTT	3.21 [2.81 to 3.68]†	2.35 [1.90 to 2.90]†	−27 [−43 to −6] %; 0.014
OGTT	2.99 [2.61 to 3.43]†	2.12 [1.72 to 2.62]†	−29 [−45 to −9] %; 0.007
**AUC_ins/glu_**			
MMTT	0.89 [0.80 to 0.97]	0.34 [0.21 to 0.48]	−0.54 [−0.71 to −0.38]; <0.001
OGTT	0.75 [0.66 to 0.84]	0.30 [0.16 to 0.44]	−0.45 [−0.61 to −0.29]; <0.001
**DI**			
MMTT	2.72 [2.57 to 2.87]	0.72 [0.49 to 0.95]	−2.01 [−2.28 to −1.73]; <0.001
OGTT	2.07 [1.92 to 2.22]	0.56 [0.33 to 0.80]	−1.51 [−1.79 to −1.23]; <0.001

*Note*: Group means with 95% CIc and between‐group differences (T2D–NGT) are shown for Matsuda ISI, AUC_ins/glu_, and DI in both test conditions.

† Estimated marginal means were calculated on the natural log scale and back‐transformed to geometric means. Percentage mean differences are expressed as percentage differences.

Abbreviations: AUC_ins/glu_, insulin secretion index; CI, confidence interval; DI, disposition index; Matsuda ISI, Matsuda insulin sensitivity index; MMTT, mixed‐meal tolerance test; NGT, normal glucose tolerance; OGTT, oral glucose tolerance test; T2D, type 2 diabetes mellitus.

### 3.3 Discriminatory ability

The Matsuda ISI showed poor discrimination (Hosmer et al., 2013) between T2D and NGT with both tolerance tests (AUROC: OGTT = 0.672, MMTT = 0.635; difference in AUROC *P* = 0.372). The AUC_ins/glu_ showed outstanding discrimination (Hosmer et al., 2013; OGTT = 0.880, MMTT = 0.897; difference in AUROC *P* = 0.498), and the DI exhibited near‐perfect discrimination (OGTT = 0.982, MMTT = 0.984; difference in AUROC *P* = 0.731). There were no statistically significant differences in AUROC between OGTT and MMTT for any index (Figure 2).

### 3.4 Reciprocal relationship between insulin sensitivity and secretion

To describe the relationship between insulin sensitivity and insulin secretion across tests and glycaemic groups, we visualized individual‐level data derived from the OGTT and MMTT (Figure 3). In both glycaemic classifications (NGT, blue; T2D, orange), both OGTT‐derived values (triangles) and MMTT‐derived values (circles) displayed the expected inversely proportionate relationship between insulin sensitivity (Matsuda ISI) and insulin secretion (AUC_ins/glu_).

### 3.5 Glucose and insulin responses by test type and glycaemic status

**FIGURE 4:**
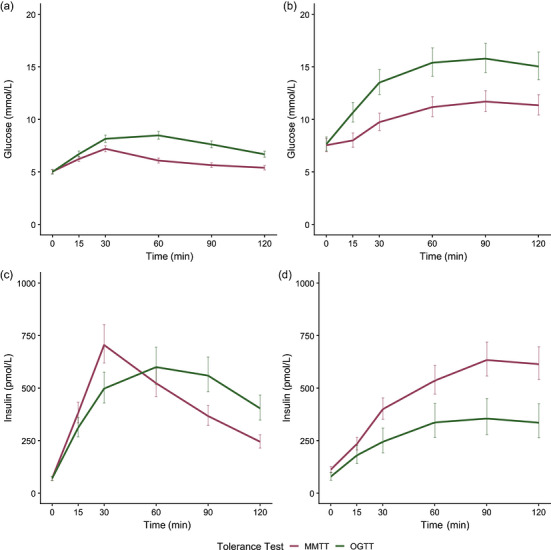
Glucose and insulin responses during OGTT and MMTT in normal glucose tolerance and type 2 diabetes. Mean (thick lines) and 95% confidence intervals (error bars) of glucose (a, b) and insulin (c, d) over 120 minutes during OGTT (green) and MMTT (purple) in participants with normal glucose tolerance (a, c) and type 2 diabetes (b, d). Abbreviations: MMTT, mixed‐meal tolerance test; OGTT, oral glucose tolerance test.

To illustrate that the OGTT and MMTT elicit distinct physiological responses, time–concentration curves showing glucose and insulin dynamics during each test (separated by glycaemic status) are displayed in Figure 4.

For glucose (Figure 4a,b), OGTT responses (green) rise more rapidly and reach higher peak values compared with MMTT responses (purple), which increase more gradually and peak at lower levels. These test‐dependent differences are evident in both NGT (difference in total AUC: *P* < 0.001) and T2D participants (difference in total AUC: *P* = 0.004), although glucose peaks occur earlier in NGT individuals.

For insulin (Figure 4c,d), NGT participants show distinct patterns; the MMTT curves display steep early‐phase peaks, whereas OGTT responses tend to rise more slowly and peak later, although overall insulin exposure appears similar between tests (difference in total AUC: *P* = 0.302). In the T2D group, the insulin response is markedly attenuated in the OGTT compared with the MMTT (difference in total AUC: *P* < 0.001), and both OGTT and MMTT insulin curves remain relatively flat.

## 4 DISCUSSION

The aim of this study was to explore potential differential metabolic responses to the OGTT and MMTT by describing the (dis)agreement between indices of insulin sensitivity, secretion and β‐cell function derived from the OGTT and MMTT. Although these tests are often interpreted interchangeably, our findings suggest that absolute values of these indices should not be considered exchangeable. This is particularly true when outcome values are high (e.g., in those with good insulin sensitivity, secretion and β‐cell function). We posit that these tests elicit different physiological responses that reflect the distinct nutrient stimuli. Interpretation of the tests should consider the potential contribution of these underlying processes.

Several studies have compared responses to the OGTT and MMTT (Chanprasertpinyo et al., 2017; Forbes et al., 2018; Marena et al., 1992; Meier et al., 2009; Wolever et al., 1998). Regardless, the existing evidence still lacks the depth to guide the optimal choice of tolerance test for a given research question or context. A few studies (Newman et al., 2022; Selimoglu et al., 2009) attempted to compare indices more directly and reported moderate‐to‐strong correlations between OGTT‐ and MMTT‐derived Matsuda ISI indices. However, we argue that these investigations do not provide enough information regarding the nature of the association to inform methodological choice. Although results from the two tests should align to some degree, absolute agreement (i.e., that the methods are exchangeable) would undermine any argument that the MMTT is more physiologically relevant. The present manuscript extends earlier efforts by providing a deeper characterization of the differences and similarities between the two challenge tests.

### 4.1 (Dis)agreement between tests

Across all investigated indices, we observed that the LoA were rather large relative to the outcome value and that they increased with the magnitude of the outcome (Figure 1). Assessment of CCC confirmed poor agreement (CCC < 0.90) for all indices (Akoglu, 2018). Despite this, there was no evidence of systematic bias between the tolerance tests. We did observe proportional biases for insulin secretion and β‐cell function, whereby the MMTT provided higher values than the OGTT as the magnitude of the outcome value increased. In other words, the average discrepancy between the two tests becomes larger in individuals with greater insulin secretion and β‐cell function. Given that the index for β‐cell function is the product of the indices for insulin sensitivity and secretion, the proportional bias in DI is likely to be driven by the proportional bias in AUC_ins/glu_.

The increased insulin secretory response observed during the MMTT could be driven by both a greater incretin response (Holst et al., 2021; Vilsbøll & Holst, 2004) and/or elevated insulin secretion in response to insulinogenic amino acids (Fajans et al., 1970; Van Loon et al., 2000; Van Sloun et al., 2020) and lipids (Mancini & Poitout, 2013). Although it is thought that β‐cell incretin sensitivity (particularly glucose‐dependent insulinotropic polypeptide) is impaired in T2D (Vilsbøll & Holst, 2004), the insulinogenic response to amino acids seems to be conserved (Nuttall et al., 1984). Furthermore, there is evidence that besides a direct insulinogenic effect of amino acids via β‐cell membrane depolarization, the presence of amino acids can stimulate incretin secretion (Nauck et al., 2021). Additionally, some fatty acids might potentiate glucose‐stimulated insulin secretion (e.g., via agonism of G protein‐coupled receptor 40), even in islets from people with T2D (Ježek, 2025; Sunil et al., 2014). Although we lack the direct evidence to test this, these specific responses to amino acids and fatty acids in the MMTT could explain why, despite the lower carbohydrate content, we observed evidence of greater insulin secretion in the MMTT (even in those with T2D). It is also possible that additional pathways, such as neural inputs (Eliasson et al., 2017; Wiedemann et al., 2022) and non‐incretin gut hormones (Cheung et al., 2009), contribute further. Interestingly, the difference between OGTT and MMTT in insulin secretion increased with higher outcome magnitudes, which might suggest that the contribution to insulin secretion from these glucose‐adjacent pathways is larger when β‐cell function is preserved. To summarize, these observations allow the possibility of an increased insulin secretory response driven by the addition of non‐glucose macronutrients in the MMTT; however, the involvement of these potential mechanisms remains speculative given the limitations of these data, and future confirmatory investigations would be required to substantiate these claims.

In both NGT and T2D participants, the MMTT produced a lower glucose AUC, which could be attributed to the lower carbohydrate content of the test, slower gastric emptying (Collier et al., 1984; Neeland et al., 2025; Shibib et al., 2024; Xiang et al., 2024) and/or altered insulin dynamics. Any volume‐related acceleration of emptying is expected only for low‐nutrient liquids, whereas in nutrient‐dense meals, such as our MMTT, small‐intestinal feedback regulation of gastric emptying dominates, making composition the more plausible driver of any potential slower gastric emptying (Doran et al., 1998). Consistent with this, we observed a faster insulin peak in the MMTT compared with the OGTT in NGT individuals, and a similar OGTT and MMTT curve shape in individuals with T2D but with a greater MMTT insulin AUC (Figure 4), in line with previous findings (Kössler et al., 2021; Rijkelijkhuizen et al., 2009; Selimoglu et al., 2009; Wopereis et al., 2017). This early peak in NGT might reflect preserved first‐phase insulin secretion and intact incretin responsiveness (Bell et al., 2015; Moghaddam et al., 2006; Shankar et al., 2016; Sheikh et al., 2024) compared with T2D; however, we remain cautious in these speculations, given the different MMTT composition between glycaemic statuses. In any case, these observations suggest that the poor agreement in indices and the differences observed in glucose and insulin curves between the MMTT and OGTT could reflect the distinct test‐specific insulin secretory stimuli.

### 4.2 Convergent validity

Although OGTT‐ and MMTT‐derived indices do not seem to agree in absolute terms, our descriptive analyses indicate that they are convergent in their assessment of the underlying physiological constructs (i.e., insulin sensitivity, insulin secretion and β‐cell function). We explored this with three descriptive analyses. First, the OGTT and MMTT both provide statistically different estimated means for Matsuda ISI, AUC_ins/glu_ and DI when comparing individuals with NGT and those with T2D (Table 2). Second, we did not observe a difference in the performance of Matsuda ISI, AUC_ins/glu_ or DI to classify individuals with NGT vs. T2D (Figure 2). Third, visual inspection of the Matsuda ISI versus AUC_ins/glu_ plot confirmed the inversely proportional relationship expected of valid indices for insulin sensitivity and secretion (i.e., for a given level of glycaemic control, insulin sensitivity and insulin secretion are complementary; Figure 3).

Although these indices are not typically used to diagnose T2D, the ROC analysis confirmed that the MMTT protocol can assess metabolic status with the same accuracy as the OGTT. We also observed that DI outperformed both the Matsuda ISI and AUC_ins/glu_ in differentiating between glycaemic classifications (Figure 2). Although this emphasizes the importance of insufficient compensatory insulin secretion in T2D, it also highlights the potential of this outcome for use in other contexts (e.g., machine learning contexts trying to identify predictors of cardiometabolic health), rather than insulin sensitivity or secretion alone. This is also illustrated in Figure 3 by the clear diagonal separation between individuals with NGT versus T2D. This observation aligns with the established reciprocal relationship between insulin sensitivity and insulin secretion (Retnakaran et al., 2008; Santos et al., 2016). These combined descriptive analyses provide evidence to support the use of the MMTT to assess insulin sensitivity, insulin secretion and β‐cell function. However, the absolute values are not directly comparable to those derived from the OGTT (i.e., they are numerically distinct), because the underlying stimuli differ. Although we believe that both the OGTT‐ and MMTT‐derived indices are valid in their own contexts, it should be stressed that they cannot be used interchangeably. The strength of the MMTT lies in capturing what could be argued as a more ecologically valid response that is likely to include the involvement of incretin hormones, glucagon and protein‐ and lipid‐induced metabolic pathways, which interact with each other and shape postprandial regulation. Therefore, if suspected mechanisms under study (e.g., treatment and/or disease pathophysiology) are thought to be mediated through altered incretin, amino‐acid or free fatty acid responses, the OGTT might not reflect such mechanisms. In this case, potentially beneficial (or harmful) interventions might be classified incorrectly unless investigated using an MMTT. In contrast, the OGTT remains a standard method for diagnosing T2D. Consequently, if the status of T2D diagnosis is important for the research question (e.g., tracking potential reversal of the condition), the OGTT would be the clear preference. Thus, the choice of test should be guided by the specific aspect of metabolism one aims to investigate.

### 4.3 Limitations

A key limitation is that indices in T2D participants were calculated from only three time points (0, 60 and 120 minutes) owing to data constraints. However, because first‐phase secretion is absent and insulin sensitivity is impaired in T2D, the resulting curves are likely to be well approximated from only these time points (Figure 4). Other important considerations are the potential for lingering effects of glucose‐lowering medication that might not have been cleared fully in the 48 hour washout period and the different MMTT compositions between NGT and T2D studies. Regarding the former, only a subset of individuals were taking medications that might still be present after 48 hour (e.g., dipeptidyl peptidase‐4 inhibitors and glucagon‐like peptide‐1 receptor agonists), but the results must be interpreted with this potential influence in mind. The different MMTT compositions are a confounder for some of the descriptive analyses supporting convergent validity; specifically, the distinct estimated means for NGT versus T2D indices. Regardless, in both NGT and T2D the MMTT‐derived indices performed in a similar manner to the OGTT regarding glycaemic status prediction and followed the expected reciprocal relationship of insulin sensitivity and secretion. As such, we believe that the bulk of the evidence is in agreement with our interpretation that the MMTT indices are valid latent indicators of the underlying physiological constructs. This was a *post hoc* cross‐sectional analysis, hence no formal sample size calculations were performed. The study would have been strengthened by a larger T2D subgroup. Furthermore, we cannot address the performance of either test in detecting longitudinal changes in these indices over time, because these analyses included only baseline assessments from the contributing studies. Another important limitation is the absence of replicate test–retest data; thus, both day‐to‐day biological variability and true variance in methodological differences contribute to these wide LoA. Finally, we did not have access to data for impaired glucose tolerance; as such, care should be taken when generalizing these findings to this population.

## 5 CONCLUSION

This *post hoc* analysis found poor absolute agreement between OGTT‐ and MMTT‐derived metabolic indices, indicated by large LoA relative to the outcome value and by increasing average bias with higher outcome values. Together, this suggests that these test‐specific indices should not be used or interpreted interchangeably. Nonetheless, both tests distinguished between NGT and T2D, showed comparable predictive ability to classify glycaemic status, and reproduced the expected inverse relationship between insulin sensitivity and secretion. Together, these findings suggest that OGTT‐ and MMTT‐derived indices demonstrate convergent validity in capturing similar underlying physiological constructs, albeit in different contexts. This underscores that test selection should be guided according to the aim; the OGTT is traditionally used for diagnosis and clinical classification, whereas the MMTT offers a more ecologically valid insight into β‐cell function and nutrient‐stimulated responses that are potentially missed by the OGTT.

## AUTHOR CONTRIBUTIONS

Conception and design: Nina Sloth Nielsen, Mathias Ried‐Larsen and Cody Garett Durrer. Data acquisition: Nina Sloth Nielsen, Helga Ellingsgaard, Kristian Karstoft, Louise Lang Lehrskov, Grit Elster Legård, Mark Preben Printz Lyngbæk, Beckey Trinh, Thomas P. J. Solomon, Marc Y. Donath and Mathias Ried‐Larsen. Data analysis and interpretation: Nina Sloth Nielsen, Nikolaj Agerlin, Nikolas Antoniou and Cody Garett Durrer. Manuscript first draft: Nina Sloth Nielsen and Cody Garett Durrer. Manuscript review: all authors. All authors read and approved the final manuscript and agree to be accountable for all aspects of the work in ensuring that questions related to the accuracy or integrity of any part of the work are appropriately investigated and resolved. All persons designated as authors qualify for authorship, and all those who qualify for authorship are listed.

## CONFLICT OF INTEREST

At the time of writing this manuscript, M.R.‐L. was an employee of Novo Nordisk A/S. There are no other potential or perceived conflicts of interest to report.

## Supporting information



Supporting Information

## Data Availability

The data used in this study are available from the corresponding author upon reasonable request.
