# Supplementary material for: (Dis)agreement and concordance of metabolic indices from the oral glucose tolerance test and mixed‐meal tolerance test: Implications for application
Source: Exp Physiol. 2026 Jun 17;111(8):3480–91. doi: 10.1113/EP093596 (PMC13394972; doi:10.1113/EP093596)
Supplement: Supplementary file 1 — Supporting Information [file EPH-111-3480-s001.docx]

**Supplemental Tables**

**Table S.1: Participant characteristics and glucose/insulin sampling schedules during OGTT and MMTT in included studies.**

| **Study DOI** | **Study number** | **Sex (F/M)** | **Age (years)** | **HbA1c (mmol/mol)** | **BMI (kg/m^2^)** | **T2D duration (years)** | **Time points MMTT** | **Time points OGTT** | **Glucose-lowering medication** |
| --- | --- | --- | --- | --- | --- | --- | --- | --- | --- |
| NGT | | | | | | | | |  |
| 10.3389/fphys.2019.01249 | 1 | 49/18 | 42.4 ± 12.8 | 35.1 ± 3.4 | 32.7 ± 9.2 | N/A | 0, 15, 30, 60, 90, 120 | 0, 15, 30, 60, 90, 120 |  |
| 10.1016/j.cmet.2018.04.008 | 2 | 0/6 | 24.0 ± 0.4 | 33.0 ± 1.1 | 24.1 ± 0.9 | N/A | 0, 15, 30, 60, 90, 120 |  |  |
| 10.1016/j.cmet.2018.04.008 | 3 | 0/7 | 24.3 ± 1.1 | 33.0 ± 2.8 | 23.9 ± 8.7 | N/A | 0, 15, 30, 60, 90, 120 | 0, 15, 30, 60, 90, 120 |  |
| 10.1007/s00125-019-05045-y | 4 | 11/6 | 47.5 ± 12.6 | 35.5 ± 3.6 | 33.4 ± 3.3 | N/A | 0, 15, 30, 60, 90, 120 |  |  |
| T2D | | | | | | | | |  |
| 10.1210/jc.2014-1837 | 5 | 3/7 | 60.3 ± 7.3 | 45.7 ± 5.9 | 28.3 ± 3.3 | 6.0 ± 2.6 | 0, 15, 30, 60, 90, 120 | 0, 60, 120 | None: 2, Metformin: 7, Sulfonylureas: 2, DPP4i: 1 |
| 10.1371/journal.pone.0163562 | 6 | 5/6 | 61.6 ± 8.3 | 46.5 ± 6.3 | 29.0 ± 5.0 | 7.0 ± 3.7 | 0, 15, 30, 60, 90, 120 | 0, 60, 120 | None: 2, Metformin: 8, Sulfonylureas: 2, DPP4i: 3 |
| 10.14814/phy2.13524 | 7 | 5/8 | 65.1 ± 7.1 | 47.8 ± 6.7 | 33.1 ± 4.0 | 8.7 ± 5.6 | 0, 15, 30, 60, 90, 120 | 0, 60, 120 | None: 2, Metformin: 11, Sulfonylureas: 3, DPP4i: 1, GLP1r agonists: 2 |
| 10.1007/s00125-014-3334-5 | 8 | 12/20 | 58.7 ± 8.0 | 49.0 ± 11.2 | 29.5 ± 4.9 | 4.6 ± 4.1 |  | 0, 15, 30, 60, 90, 120 | None: 13, Metformin: 17, Sulfonylureas: 7, DPP4i: 2, GLP1r agonists: 3 |

**Data are presented as means ± standard deviations.**

**Table S.2: Composition of MMTT beverages used across the included studies**

| **Study DOI** | **Study number** | **Energy (kcal)** | **Volume (ml)** | **Carbohydrate (% energy)** | **Fat (% energy)** | **Protein (% energy)** |
| --- | --- | --- | --- | --- | --- | --- |
| NGT | | | | | | |
| 10.3389/fphys.2019.01249 | 1 | 385 | 360 | 65 | 15 | 20 |
| 10.1016/j.cmet.2018.04.008 | 2 | 385 | 360 | 65 | 15 | 20 |
| 10.1016/j.cmet.2018.04.008 | 3 | 385 | 360 | 65 | 15 | 20 |
| 10.1007/s00125-019-05045-y | 4 | 362 | 360 | 68 | 15 | 17 |
| T2D | | | | | | |
| 10.1210/jc.2014-1837 | 5 | 450 | 300 | 55 | 30 | 15 |
| 10.1371/journal.pone.0163562 | 6 | 450 | 300 | 55 | 30 | 15 |
| 10.14814/phy2.13524 | 7 | 450 | 300 | 55 | 30 | 15 |
